# Rabies post-exposure healthcare-seeking behaviors and perceptions: Results from a knowledge, attitudes, and practices survey, Uganda, 2013

**DOI:** 10.1371/journal.pone.0251702

**Published:** 2021-06-02

**Authors:** Sarah C. Bonaparte, Laura Adams, Barnabas Bakamutumaho, Galileu Barbosa Costa, Julie M. Cleaton, Amy T. Gilbert, Modupe Osinubi, Emily G. Pieracci, Sergio Recuenco, Victor Tugumizemu, Joseph Wamala, Ryan M. Wallace

**Affiliations:** 1 Poxvirus and Rabies Branch, Division of High-Consequence Pathogens and Pathology, National Center of Emerging and Zoonotic Infectious Diseases, United States Centers for Disease Control and Prevention, Atlanta, Georgia, United States of America; 2 ORISE Fellow, United States Centers for Disease Control and Prevention, Atlanta, Georgia, United States of America; 3 Epidemic Intelligence Service, Centers for Disease Control and Prevention, Atlanta, Georgia, United States of America; 4 Uganda Virus Research Institute, Kampala, Uganda; 5 Veterinary Public Health Division, Ministry of Health, Kampala, Uganda; 6 World Health Organization, Kampala, Uganda; University of Pretoria, SOUTH AFRICA

## Abstract

**Background:**

Rabies is a viral disease of animals and people causing fatal encephalomyelitis if left untreated. Although effective pre- and post-exposure vaccines exist, they are not widely available in many endemic countries within Africa. Since many individuals in these countries remain at risk of infection, post-exposure healthcare-seeking behaviors are crucial in preventing infection and warrant examination.

**Methodology:**

A rabies knowledge, attitudes, and practices survey was conducted at 24 geographically diverse sites in Uganda during 2013 to capture information on knowledge concerning the disease, response to potential exposure events, and vaccination practices. Characteristics of the surveyed population and of the canine-bite victim sub-population were described. Post-exposure healthcare-seeking behaviors of canine-bite victims were examined and compared to the related healthcare-seeking attitudes of non-bite victim respondents. Wealth scores were calculated for each household, rabies knowledge was scored for each non-bitten survey respondent, and rabies exposure risk was scored for each bite victim. Logistic regression was used to determine the independent associations between different variables and healthcare-seeking behaviors among canine-bite victims as well as attitudes of non-bitten study respondents.

**Results:**

A total of 798 households were interviewed, capturing 100 canine-bite victims and a bite incidence of 2.3 per 100 person-years. Over half of bite victims actively sought medical treatment (56%), though very few received rabies post-exposure prophylaxis (3%). Bite victims who did not know or report the closest location where PEP could be received were less likely to seek medical care (p = 0.05). Respondents who did not report having been bitten by a dog with higher knowledge scores were more likely to respond that they would both seek medical care (p = 0.00) and receive PEP (p = 0.06) after a potential rabies exposure event.

**Conclusions:**

There was varying discordance between what respondents who did not report having been bitten by a dog said they would do if bitten by a dog when compared to the behaviors exhibited by canine-bite victims captured in the KAP survey. Bite victims seldom elected to wash their wound or receive PEP. Having lower rabies knowledge was a barrier to theoretically seeking care and receiving PEP among not bitten respondents, indicating a need for effective and robust educational programs in the country.

## Introduction

Rabies virus (RABV) is a *Lyssavirus* which causes encephalitic disease known commonly as rabies [1]. While cases of human rabies caused by other *lyssaviruses* have been reported, fatalities resulting from these encephalitides are very rare [2]. If left untreated, RABV encephalomyelitis is nearly 100% fatal [3]. The canine rabies virus variant (CRVV) is the most common cause of human infections and is responsible for nearly 99% of human deaths due to rabies [4]. Controlling the disease in canine populations, especially free-roaming dog populations, is the most effective method to prevent human rabies deaths [5]. Established methods of dog population management and vaccination have been successful in eliminating canine-mediated rabies in most of the Western hemisphere, Western Europe, and some Asian countries [6].

The global burden of rabies remains substantial, causing an estimated 59,000 deaths annually [4]. While a reduction in human rabies deaths has been actualized in many locations from controlling the disease in canine populations [6], deaths still occur in numerous countries due to lack of disease surveillance, barriers to accessing appropriate post-exposure prophylaxis (PEP), and under-appreciation of the public health implications of the disease [7]. The populations most affected tend to be in highly impoverished countries, and harmful economic impacts which result burden these communities [4]. Africa alone experiences 36.4% of the global burden of human rabies deaths, and the poorest countries in sub-Saharan Africa are burdened with the highest per-person death rate due to rabies [4]. These high mortality rates result from limited dog vaccination and inadequate PEP supply and distribution [4]. With no routine laboratory surveillance diagnostic capacity available within the country [8] and dogs inflicting 94% of animal bites in the country [9], Uganda’s rabies burden is of considerable concern.

A previous study utilized results from a knowledge, attitudes, and practices (KAP) survey to estimate dog ownership and RABV vaccination coverage and to identify barriers to effective dog vaccination throughout Uganda [10]. This study found that wealthier communities claimed ownership of more dogs and reported higher dog vaccination rates than less wealthy communities [10]. Though findings from that study could be used to design and implement methods of mass canine rabies vaccination, an estimated 26 million individuals remain at risk of exposure to rabies in Uganda [10]. Until large-scale, effective mass vaccination programs are implemented in Uganda, post-exposure healthcare seeking behaviors will remain crucial life or death judgements for these individuals. While no studies have analyzed healthcare-seeking behaviors for people exposed to rabies in Uganda, a recent KAP study from Cameroon identified that increased wealth and knowledge were associated with a survey respondent seeking healthcare and receiving post-exposure prophylaxis after a canine-bite [11]. This study uses the Uganda KAP survey to explore the post-bite healthcare-seeking behaviors of Ugandans and identify factors that influence or prevent appropriate exposure treatment. The specific aims of this study are to a) compare healthcare-seeking behaviors of canine-bite victims with the healthcare perceptions of not-bitten survey respondents, b) identify factors that are associated with, and may represent barriers to, recommended post-exposure healthcare-seeking behaviors among canine-bite victims, and c) identify factors that are associated with perceptions about post-exposure healthcare-seeking behaviors that may reveal avenues for improvement in future behaviors.

## Methods

### Survey and study population

A cross-sectional study in the form of a KAP survey was conducted in Uganda during 2013 to capture information on dog ownership, knowledge concerning rabies disease, responses to potential or actual bite exposure events, and vaccination practices (S1 Appendix). Multi-stage cluster sampling was used to identify 25 different sites within the country. Five districts in Uganda were selected based on representative location within the country and presence of bite incident surveillance infrastructures. Within each of the selected districts, five villages were selected using a random number generator and skip patterns to ensure an even distribution throughout the district. Individual residences within the villages were selected by approaching every other, every third, or every fourth household in a counting series along mapped routes, depending on the estimated number of households per community. If no respondent answered the door or the respondent did not give consent to be interviewed, the surveyors continued to the following house in the skip pattern and household was not included in the survey. Interview data was collected using handheld personal digital assistant devices (PDAs) which also recorded the GPS coordinates of each respondent’s residence. A map of the surveyed locations was created using ArcMAP 10.5 (ESRI, Redlands, CA). Written informed consent was collected and these consenting respondents received a bar of soap as well as rabies-prevention educational materials that emphasized the importance of post-bite wound washing with soap and water. Interviewers requested to speak with the head of household at each residence, but other respondents were eligible for participation if they were at least 18 years of age and the head of household was not available [10]. Based on a population of 4,687,081 (http://www.statoids.com/uug.html) among the five districts represented and confidence level of 95%, we calculated a minimum sample size of 384. The number of houses approached and interviewed beyond the minimum required sample size was based on funds available, timeline allocated for the study, and number of household representatives which consented to be interviewed. This study was approved by the Centers for Disease Control and Prevention’s Human Research Office, protocol number 6312, and the Uganda Virus Research Institute, reference number GC/127/12/11/28.

**Fig 1:**
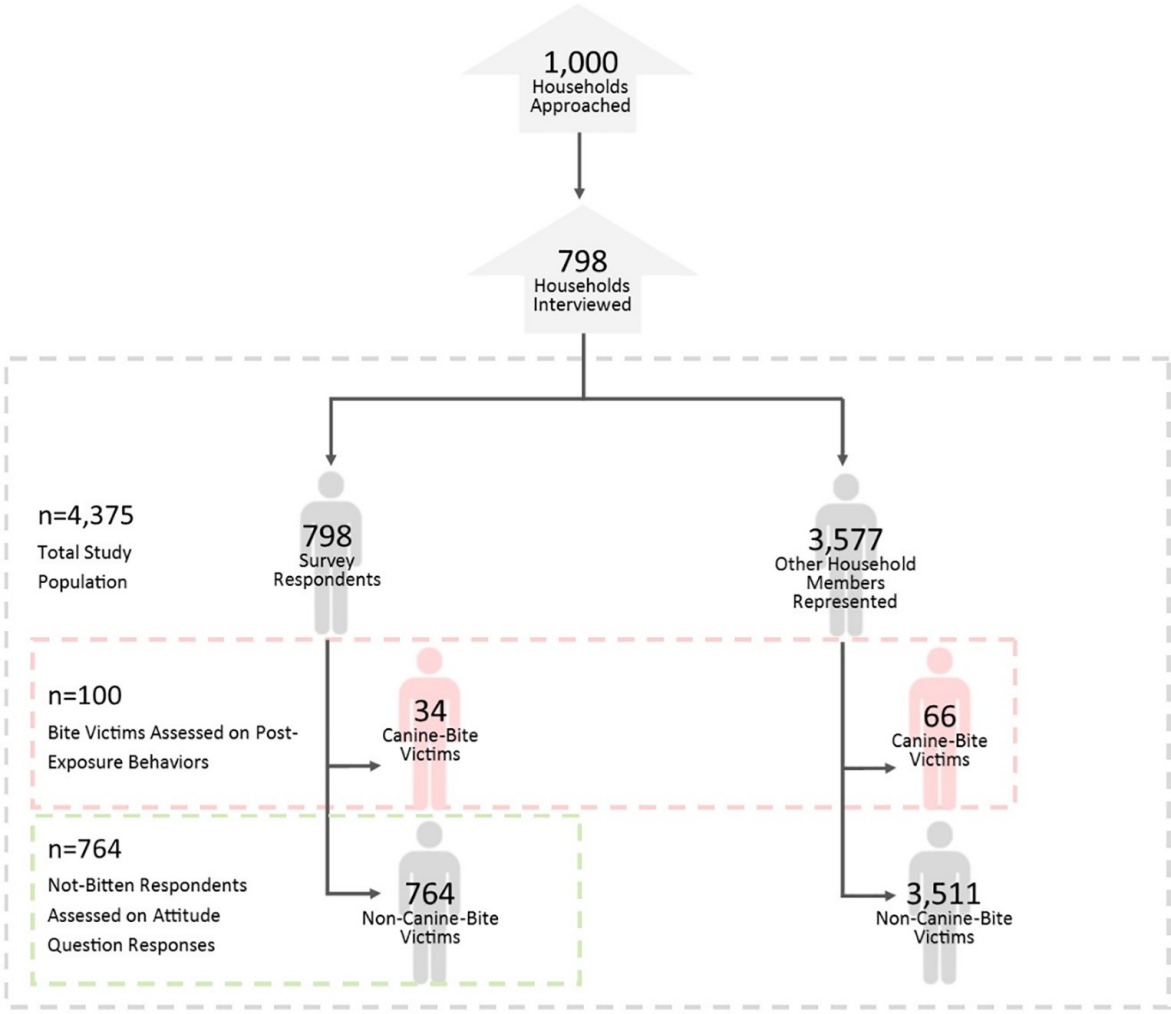
Study population by analysis groups.

Questions posed by the interviewer gathered information about the respondent, the respondent’s household, or anyone living within the residence. Major categories of questions included participant demographic information, household characteristics, animals owned by the household, dog bite information, domestic and wild animal bite information, and rabies knowledge of the respondent. Within the surveyed population, outcomes were analyzed in two sub-populations (Fig 1). First, if respondents reported that they or anyone living at their residence had been bitten by a dog in the past year, these respondents and household members were classified as ‘bite victims’ and asked about their actions after being bitten. Second, if respondents did not report having been bitten by a dog within the past year, they were classified as ‘not bitten respondents’ and were asked about what they would do if bitten.

### Wealth, knowledge, and bite scoring

Wealth scores were calculated to evaluate the wealth of each responding household (Table 1). Certain questions in the KAP survey were designed to assess the education level of the respondent and the number and type of livestock owned by the respondent’s household. Cutoffs for education level were determined by the structure of the education system in Uganda, consisting of seven years of primary school, six years of secondary school, and university or tertiary school following (https://www.theguardian.com/katine/2010/feb/08/education-system-explainer). Value of livestock was extrapolated from “Africa Farming” online magazine (http://africafarming.info/how-much-does-an-animal-cost/). Quality of domicile construction was determined from the evaluation of doors, windows, floors, walls, and roofs of the residences by the surveyors (S2 Table). A combination of points, with a maximum of 10 points, were rewarded or deducted for each of the three categories assessed.

**Table 1:** Construction of wealth scores.

Variables used to assess the wealth scores		
**Years of schooling completed**	**Education level**	**Points assigned**
0	None	-10
1–7	Low	-3.3
8–13	Medium	+3.3
> 13	High	+10
**Construction of house^a^**	**Quality level**	**Points assigned**
Minimal^b^	Poor	-10
Dirt/sand/straw/wood	Low	-3.3
Cement/brick/wood/metal	Medium	+3.3
Tile/cement/glass/metal	High	+10
**Livestock owned^c^**	**Commercial value/head (USD)**	**Points assigned^d^**
Cattle	$500	
Pigs	$150	-10 ($0)
Sheep	$120	-3.3 ($1 - $199)
Goats	$80	+3.3 ($200 - $999)
Turkeys	$16	+10 (>$999)
Fowl, Rabbits, Guinea Pigs	$8	
Pigeons	$4	

^a^Responses described by survey assistant, not survey respondent, based on evaluation of doors, windows, floor, walls, and roof of residence

^b^Minimal construction of house indicates missing doors and/or windows

^c^Multiple responses allowed on questionnaire

^d^Points assigned based on summary of all livestock owned per household

Knowledge scores were calculated to evaluate the respondent’s knowledge about rabies disease severity, transmission, infection, and treatment from various open- and close-ended questions (Table 2). A combination of points, with a maximum of 10 points, were rewarded or deducted for each of the four categories assessed.

**Table 2:** Construction of knowledge scores.

Questions used to assess knowledge scores	First preferred answer (points assigned)	Second preferred answer (points assigned)	Incorrect answers (points assigned)^e^
How severe is the disease called rabies?	Very severe (10 pts)		Mild (-10 pts) Somewhat severe (-10 pts)
How do humans get rabies from an infected animal?^a^	Bite (3 pts) Contact with saliva (3 pts)	Scratch (2 pts) Other, correct responses^b^ (2 pts)	Observing the animal (-2 pts) Touching the animal (-2 pts) Contact with blood (-2 pts) Contact with urine/feces (-2 pts) Other, incorrect responses^b^ (-2 pts)
What animals can be infected with rabies?^a^	*Local Reservoir Species*: • Dogs (2 pts) • Bats (2 pts) • Jackals (2 pts)	*Non-Reservoir*, *but Susceptible*: • Cats (0.5 pts) • Livestock^c^ (0.5 pts) • Horses (0.5 pts) • Hyenas (0.5 pts) • Mongooses (0.5 pts) • Monkeys or other primates (0.5 pts) • Foxes (0.5 pts) • Rodents (0.5 pts)	Wild birds (-5 pts) Poultry^d^ (-5 pts) Other responses^b^ (0 pts)
If you thought that you had an exposure to an animal with rabies, what would you do?^a^	Wash wound (2.6 pts) Actively seek medical treatment at a pharmacy, hospital, clinic or outpost (2.6 points) Receive rabies post-exposure prophylaxis (2.6 points)	Isolate the animal for observation (1.1 points) Submit animal for disease testing (1.1 points)	Consult with a traditional healer (-2.5 pts) Call a medical doctor (0 pts) Call a veterinarian (0 pts) Kill the animal (0 pts) Kill and eat the animal (-2.5 pts) Nothing (-5 pts) Other responses^b^ (0 pts)

^a^Multiple and/or free responses allowed

^b^Based on the option ‘Other’ allowing free response answers for multiple response questions

^c^Livestock includes cattle, sheep, goats, etc.

^d^Poultry includes chickens, ducks, geese, etc.

^e^Responses indicating that they did not know the answer were awarded 0 points

Rabies exposure risk scores were created for each bite victim to estimate the level of hazard for reported canine-bites (Table 3). Risk rank score was calculated as the sum of points based on four bite variables: most severe anatomical location of the bite, bite frequency, whether the bite was provoked, and familiarity with the dog. Based on the *a priori* determination of risk levels, a rabies exposure risk score of 1–6 was considered ‘low’ risk, a score of 7–9 was considered ‘medium’ risk, and a score of 10–12 was considered ‘high’ risk.

**Table 3:** Construction of rabies exposure risk scores.

Points Assigned	Bite Location^a^	Bite Severity^b^	Bite Activity^c^	Familiarity with Dog
0	Missing/declined to answer/unknown		Missing/declined to answer	Missing/declined to answer/unknown
1	Torso/arms/legs	Single	Provoked	Own dog
2	Hands/feet	Not reported	Unprovoked/interacting with the dog	Neighbor’s dog/Dog in community
3	Head/face	Multiple	Unprovoked/avoiding the dog	Did not recognize dog

^a^Multiple anatomical Bite Locations may have been reported, most severe location of bite used for scoring

^b^Bite Severity correlates to the number of bites reported per bite event

^c^Bite Activity correlates to the behavior of the individual at the time they were bitten

### Covariates of interest and statistical analysis

Due to the format of the survey, ‘other’ free response answers to various multiple-response questions were frequent (S1 Appendix). These responses were re-categorized to the most appropriate categorical response presented by the surveyor or were assigned a new response category if multiple participants responded similarly. Community bite rate was calculated for each village as the sum of all reported dog bites among household members divided by the total number of household members reflected in the surveys. Percent below poverty was defined as the percent of people in 2013 living below the international poverty line of 1.25 U.S. dollars per day [12]. Data were modified for variables with less than 5% missing responses for exposures used in regression models according to the rationale of a respondent’s answers to other survey questions or using the village average of the variable.

Exposures of interest for a given sub-population included covariates that could affect heath care-seeking behaviors after a potential rabies exposure. These independent variables were selected based on data available from the KAP survey, disease subject matter expertise, and Bradford Hill Criteria qualification [13]. For not bitten respondents, these variables included age, sex, percent below poverty, district, status of dog ownership, nearest distance to PEP, knowledge score, and wealth score. The effect of the interaction of the wealth and knowledge score variables was assessed but not significant, and therefore was not included in further analysis. For bite victims, these variables included age, sex, percent below poverty, district, ownership of the biting dog, nearest distance to PEP, rabies exposure risk score, and wealth score. Of these predictors, community-level variables included percent below poverty and district. Wealth score was calculated to compare wealth status between households while percent below poverty marks overall community poverty in relation to the entire country.

Outcomes of interest for both sub-populations in this analysis included two crucial post-canine-bite event healthcare-seeking behaviors: actively seeking medical treatment and receiving PEP. These favorable behaviors are included in the World Health Organization’s (WHO) post-exposure treatment guidelines of washing the wound, actively seeking medical attention, and receiving PEP [14]. The adverse outcome of seeking traditional medicine or traditional medical treatments was also examined for the bite victim cohort. Due to the low number of outcomes, receiving PEP was not able to be modelled for the bite victim cohort. These outcomes were not mutually exclusive in the survey questionnaire.

Demographic characteristics of the entire survey population and of bite victims were described. Healthcare-seeking behaviors of bite victims were compared to perceptions of post-bite care as reported by non-bitten respondents. One-way Analysis of Variance (ANOVA) compared average household wealth and knowledge scores between districts and villages. Several multivariable logistic regression models were constructed to examine associations between the variables previously identified as exposures of interest and particular healthcare-seeking behaviors. Adjusted odds ratios (aORs) with corresponding 95% confidence intervals were reported using p-values < 0.1 conservatively considered to indicate a significant association. Microsoft Excel (Microsoft Corporation, Redmond, Washington, USA) was used for data cleaning, SAS v9.4 (SAS Institute, Cary, North Carolina, USA) was used for descriptive data analysis, and EpiInfo v7.2 (http://www.cdc.gov/epiinfo/) was used for logistic regression modelling.

## Results

### Study population

**Fig 2:**
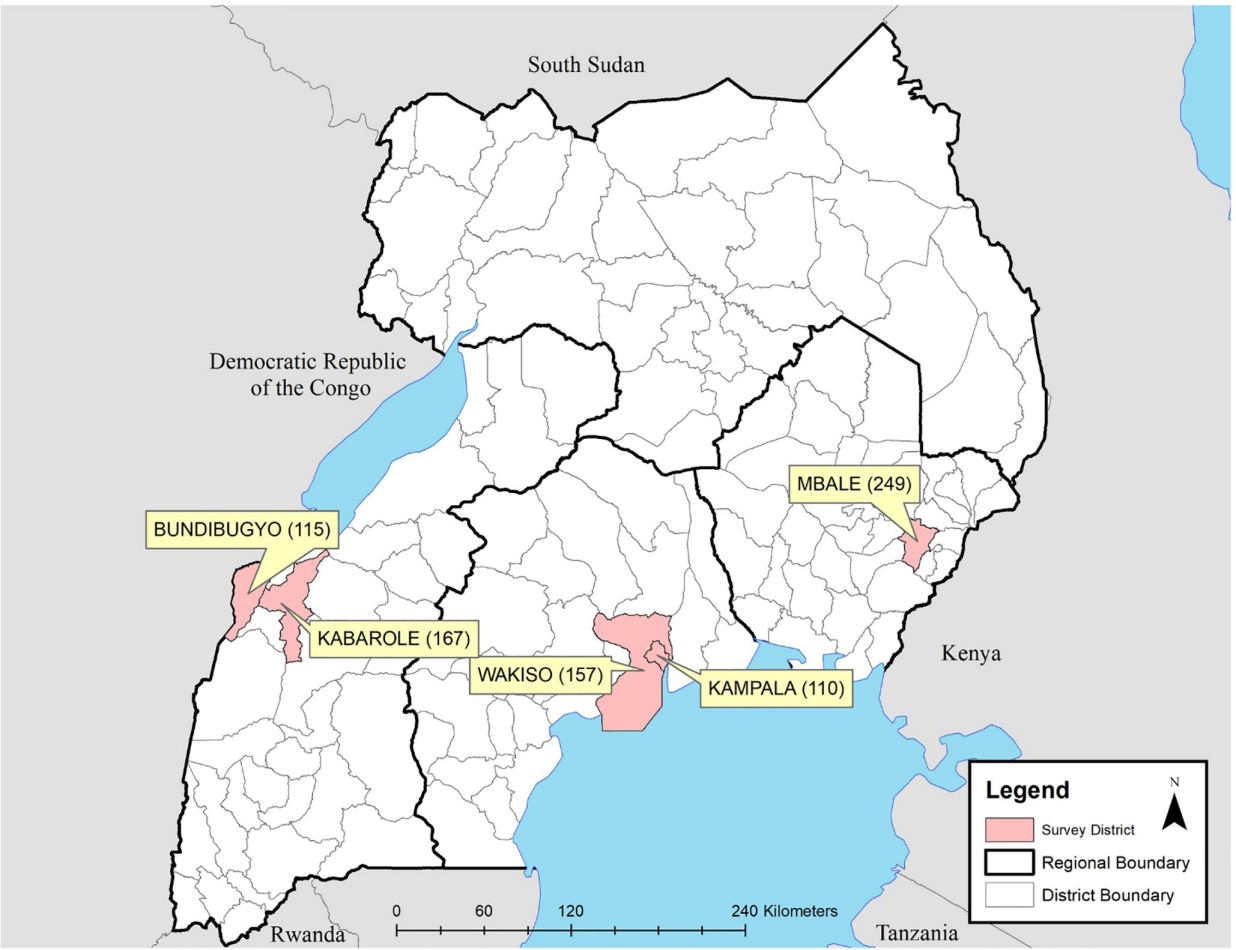
Districts and number of households surveyed in Uganda. Administrative boundaries were obtained from www.gadm.org.

**Fig 3:**
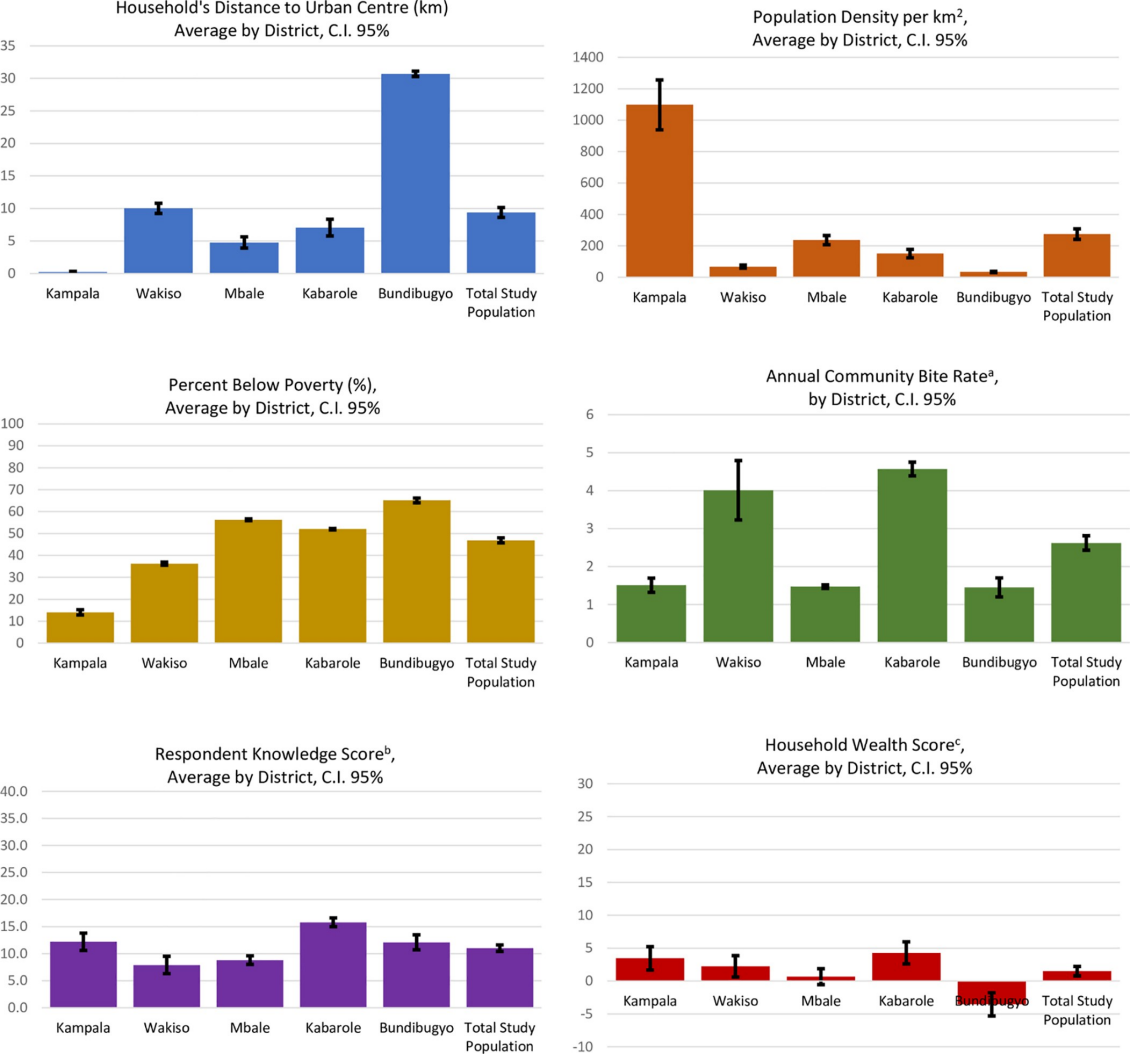
Characteristics of the five surveyed districts, Uganda, 2013. ^a^ Rate per 100 people. ^b^Household wealth scored on a scale of -40 to 40. ^c^ Rabies knowledge scored on a scale of -30 to 30.

The selected districts were previously described by Wallace et al, and included Kampala, Wakiso, Mbale, Kabarole, and Bundibugyo (Fig 2) [10]. Out of the 25 selected villages, one village in Bundibugyo was not able to be surveyed. Among the five districts, 798 households completed the survey out of 1,000 that were approached, representing 4,375 household members (Fig 1). The 24 villages represented varied greatly in demographic characteristics such as population density (range 2–1,401 km^2^) (http://www.citypopulation.de/Uganda-Cities.html?cityid=1762) and percent below poverty (range 5.7–74.5%) (http://www.worldpop.org.uk/data/summary/?doi=10.5258/SOTON/WP00285, http://www.unicef.org/infobycountry/uganda_statistics.html) [10]. The districts’ average wealth and knowledge scores had significantly different means (Fig 3).

### Attitudes concerning post-rabies exposure healthcare-seeking behaviors

Of the 798 survey respondents, 764 (95.7%) reported that they were not bitten by a dog within the past year (Table 4). Among these not bitten respondents who answered questions regarding what they would have done if they thought they had an exposure to an animal with rabies, 6 (0.8%) responded that they would have followed the complete WHO guidelines of washing their wound, actively seeking medical care, and receiving PEP [14]. The majority (69.6%) of not bitten respondents said they would seek medical attention, while less than 5% of not bitten respondents said they would either wash their wound or receive PEP. Few not bitten respondents reported unfavorable responses, with 1.6% indicating they would consult with a traditional healer and 2.1% indicating they would do nothing if bitten. There were no statistical differences in the four main attitudes towards bites (washing the wound, consulting with a traditional healer, seeking medical care, and receiving PEP) between not bitten respondents in urban, semi-urban, and rural locations by chi-square testing.

**Table 4:** Attitudes and practices concerning post-bite healthcare-seeking behavior categories among community members, Uganda, 2013.

Attitudes towards bites from suspected rabid animals by respondents who had not been bitten n = 764^a^		Practices of canine-bite victims n = 100	
	*n (%)*		*n (%)*
If you thought you had an exposure to an animal with rabies what would you do?^b^		What did you/they do when bitten by the dog?^b^	
Wash the wound	37 (4.8%)	Washed the wound	9 (9.0%)
Consult with a traditional healer	12 (1.6%)	Consulted with a traditional healer	21 (21.0%)
Call a medical doctor	53 (6.9%)	Called a medical doctor	0 (0.0%)
Call a veterinarian	40 (5.2%)	Called a veterinarian	3 (3.0%)
Actively seek medical treatment	532 (69.6%)	Actively sought medical treatment	56 (56.0%)
Receive rabies PEP	24 (3.1%)	Received rabies PEP	3 (3.0%)
Isolate the animal for observation	8 (1.0%)	Isolated the dog for observation	0 (0.0%)
Submit the animal for rabies testing	5 (0.7%)	Submitted the dog for rabies testing	0 (0.0%)
Kill the animal	49 (6.4%)	Killed the dog	2 (2.0%)
Nothing	16 (2.1%)	Nothing	13 (13.0%)

^a^Respondents who had not been bitten includes all survey respondents excluding those who reported being bitten by a dog

^b^Multiple responses allowed, column totals may not add up to 100%

Among not bitten respondents, actively seeking medical care was significantly associated with living in Mbale (aOR = 2.05, 95% C.I. 1.05, 4.00), living 6–20 (aOR = 2.59, 95% C.I. 1.45, 4.63) or greater than 20 (aOR = 3.11, 95% C.I. 1.53, 6.31) kilometers from a location where PEP could be received, not knowing or not reporting nearest distance from a location where PEP could be received (aOR = 0.30, 95% C.I. 0.18, 0.51), and increased knowledge score (aOR = 1.12, 95% C.I. 1.09, 1.15; (Table 5)). Additionally, seeking PEP was significantly associated with increased age (aOR = 1.03, 95% C.I. 1.00, 1.05) and increased knowledge score (aOR = 1.08, 95% C.I. 1.00, 1.17) for this sub-population. While age (aOR = 0.99, 95% C.I. 0.98, 1.00), living in Kabarole (aOR = 0.51, 95% C.I. 0.26, 1.02), and owning a dog (aOR = 1.78, 95% C.I. 0.96, 3.30) showed p-values less than the cutoff of 0.1 in the first model, the range in confidence intervals prevents the association from being considered significant.

**Table 5:** Characteristics associated with attitudes concerning healthcare-seeking behaviors among 764 not bitten respondents, Uganda, 2013.

		Outcome: Would seek medical care (n = 530)		Outcome: Would receive PEP (n = 24)	
Variables in model	n^a^	aOR (95% C.I.)	p-value	aOR (95% C.I.)	p-value
**Age**	36.3	0.99 (0.98–1.00)	0.03	1.03 (1.00–1.05)	**0.06**
**Sex**					
Male	250	ref		ref	
Female	514	0.77 (0.51–1.17)	0.22	0.60 (0.24–1.50)	0.27
**% below poverty**	46.1	0.99 (0.96–1.02)	0.45	0.96 (0.90–1.02)	0.22
**District**					
Bundibugyo	113	ref		ref	
Kabarole	153	0.51 (0.26–1.02)	0.06	2.38 (0.46–12.21)	0.30
Kampala	106	0.44 (0.11–1.76)	0.24	0.91 (0.05–17.02)	0.95
Mbale	243	2.05 (1.05–4.00)	**0.04**	1. 20 (0.17–8.41)	0.86
Wakiso	149	1.17 (0.46–2.97)	0.75	0.00 (0.00 - >1.0E12)	0.97
**Dog owner (Yes)**	98	1.78 (0.96–3.30)	0.07	1.49 (0.50–4.45)	0.48
**Distance to PEP**					
0–5 km	338	ref		ref	
6–20 km	145	2.59 (1.45–4.62)	**0.001**	1.77 (0.64–4.88)	0.27
>20 km	114	3.11 (1.53–6.31)	**0.002**	0.24 (0.03–2.03)	0.19
Unknown/Unreported	167	0.30 (0.18–0.51)	**0.0000**	0.48 (0.05–4.22)	0.50
**Knowledge score**	11	1.12 (1.09–1.15)	**0.0000**	1.08 (1.00–1.17)	**0.06**
**Wealth score**	1.5	1.00 (0.98–1.02)	0.98	1.03 (0.99–1.08)	0.15

aOR, adjusted odds ratio; C.I., confidence interval

^a^Averages for continuous variables and counts otherwise

### Post-rabies exposure responses of canine-bite victims

One hundred canine-bite victims were observed among the survey population of 4,375 individuals (range 0–16 bites per village), yielding an annual average community bite rate of 2.3 per 100 people (S1 Table). The rate of dog bites in the youngest age category (0–15 years) was 1.37 times higher than the rest of the bite victim sub-population; an expected outcome since children under 15 years have the highest rabies exposure risk worldwide [14–16]. Eighty-six (86%) bite victims were bitten by dogs that they owned or recognized and 13 (13%) were bitten by dogs that they did not recognize (Table 6). Eighteen (18%) canine-bites were categorized as ‘high’ rabies exposure risk, while 65 (65%) were categorized as ‘medium’ and 17 (17%) were categorized as ‘low’ rabies exposure risk (Table 3). Only 1 victim reported being bitten on the head while the majority reported being bitten on their torso, arms, or legs. There were no statistical differences in bite rates or in the four main healthcare-seeking behaviors (washing the wound, consulting with a traditional healer, seeking medical care, and receiving PEP) between bite victims in urban, semi-urban, and rural surveyed locations by chi-square testing.

**Table 6:** Characteristics and healthcare-seeking behaviors of 100 canine-bite victims among the 798 surveyed households, Uganda, 2013.

Characteristics	All Victims N = 100 *n (%)*	Favorable Behaviors per WHO Tretment Guidelines^a^			Unfavorable Behaviors^a^	
		Washed their Wound n = 9 *n (%)*^b^	Actively Sought Medical Treatment n = 56 *n (%)*^b^	Received PEP n = 3 *n (%)*^b^	Employed Traditional Medicine n = 21 *n (%)*^b^	Did Nothing Once Bitten n = 13 *n (%)*^b^
**Age (years)**						
0–15	59 (59.0%)	8 (13.6%)	35 (59.3%)	2 (3.4%)	14 (23.7%)	6 (10.2%)
16–30	22 (22.0%)	0 (0.0%)	13 (59.1%)	1 (4.5%)	1 (4.5%)	3 (13.6%)
31–45	12 (12.0%)	0 (0.0%)	3 (25.0%)	0 (0.0%)	5 (41.7%)	3 (25.0%)
>45	7 (7.0%)	1 (14.3%)	5 (71.4%)	0 (0.0%)	1 (14.3%)	1 (14.3%)
**Sex**						
Male	52 (52.0%)	4 (7.7%)	32 (61.5%)	2 (3.8%)	11 (21.2%)	5 (9.6%)
Female	48 (48.0%)	5 (10.4%)	24 (50.0%)	1 (2.1%)	10 (20.8%)	8 (16.7%)
**District**						
Kampala	8 (8.0%)	0 (0.0%)	4 (50.0%)	0 (0.0%)	3 (37.5%)	1 (12.5%)
Wakiso	18 (18.0%)	1 (0.6%)	11 (61.1%)	0 (0.0%)	6 (33.3%)	0 (0.0%)
Mbale	20 (20.0%)	1 (0.05%)	13 (65.0%)	1 (0.05%)	0 (0.0%)	5 (25.0%)
Kabarole	43 (43.0%)	7 (16.3%)	20 (46.5%)	2 (4.7%)	8 (18.6%)	7 (16.3%)
Bundibugyo	11 (11.0%)	0 (0.0%)	8 (72.7%)	0 (0.0%)	4 (36.4%)	1 (9.1%)
**Percent below poverty level**^**b**^						
0–15%	4 (4.0%)	0 (0.0%)	1 (25.0%)	0 (0.0%)	2 (50.0%)	1 (25.0%)
16–35%	8 (13.0%)	1 (12.5%)	4 (50.0%)	0 (0.0%)	4 (50.0%)	0 (0.0%)
36–55%	60 (60.0%)	6 (10.0%)	33 (55.0%)	1 (1.7%)	8 (13.3%)	10 (16.7%)
>55%	29 (23.0%)	2 (6.9%)	18 (62.1%)	2 (6.9%)	7 (24.1%)	2 (6.9%)
**Perceived distance from nearest location where rabies PEP could be received**						
0–5 km	53 (53.0%)	4 (7.5%)	31 (58.5%)	0 (0.0%)	11 (20.8%)	5 (9.4%)
6–20 km	22 (22.0%)	4 (18.2%)	13 (59.1%)	1 (4.5%)	3 (13.6%)	5 (22.7%)
> 20 km	8 (8.0%)	0 (0.0%)	3 (37.5%)	1 (12.5%)	4 (50.0%)	1 (12.5%)
Unreported/Unknown	17 (17.0%)	1 (5.9%)	9 (52.9%)	1 (5.9%)	3 (17.6%)	2 (11.8%)
**Ownership of biting dog**						
Own/Family Dog	9 (9.0%)	0 (0.0%)	5 (55.6%)	1 (11.1%)	3 (33.3%)	1 (11.1%)
Neighbor’s Dog	44 (44.0%)	5 (11.4%)	24 (54.5%)	1 (2.3%)	10 (22.7%)	5 (11.4%)
Dog in Community	33 (33.0%)	1 (3.0%)	20 (60.6%)	1 (3.0%)	7 (21.2%)	4 (12.1%)
Unrecognized Dog	13 (13.0%)	3 (23.1%)	7 (53.8%)	0 (0.0%)	1 (7.7%)	3 (23.1%)
Unreported	1^c^ (1.0%)	0 (0.0%)	0 (0.0%)	0 (0.0%)	0 (0.0%)	0 (0.0%)
**Potential bite outcome**^**d**^						
Healthy, No Illness	81 (81.0%)	7 (8.6%)	45 (55.5%)	3 (3.7%)	17 (21.0%)	11 (13.6%)
Illness	19 (19.0%)	2 (10.5%)	11 (57.9%)	0 (0.0%)	4 (21.1%)	2 (10.5%)
Death from Illness	5 (5.0%)	0 (0.0%)	4 (80.0%)	0 (0.0%)	0 (0.0%)	1 (20.0%)
**Rabies exposure risk**^**e**^						
Low	17 (17.0%)	1 (5.9%)	10 (58.8%)	1 (5.9%)	2 (11.8%)	2 (11.8%)
Medium	65 (65.0%)	5 (7.7%)	37 (56.9%)	1 (1.5%)	15 (23.1%)	9 (13.8%)
High	18 (18.0%)	3 (16.7%)	9 (50.0%)	1 (5.6%)	4 (22.2%)	2 (11.1%)

^a^Multiple responses allowed, totals may not add up to 100%

^b^Community level variable

^c^The bite victim who did not report ownership of the biting dog did not report any favorable or unfavorable bite response behaviors

^d^Response ‘Healthy’ mutually exclusive from responses ‘Illness’ and ‘Death,’ ‘Illness’ and ‘Death’ are not mutually exclusive

^e^Rabies Exposure Risk Rank Score categories are: Low = 1–5 total points, Medium = 6–10 total points, High = 11–15 total points.

More than half (56%) of bite victims reported that they actively sought medical treatment while only 3 (3%) reported receiving PEP. None of the bite victims followed all of the World Health Organization recommendations for rabies post-exposure treatment (i.e., wash the wound, seek medical care, and initiate PEP). Twenty-one percent of victims reported that they sought or employed traditional medicine, though 3 (14%) of these individuals also enacted another healthcare-seeking behavior. One of these bite victims who employed traditional medicine also washed their wound, one also sought medical care, and the third victim washed their wound and sought care. Among the canine-bite victims, 19 (19%) were reported to have fallen ill after being bitten with 5 resulting fatalities, though these fatalities cannot definitely be contributed to CRVV infection. Of the 5 individuals who died, 4 actively sought treatment, but none received PEP. Of the 13 bite victims who did nothing after having been bitten, 2 fell ill and 1 died. None of the three individuals who received PEP died after having been bitten.

Among bite victims, not knowing or not reporting nearest distance from a location where PEP could be received (aOR = 0.18, 95% C.I. 0.03, 0.99) was negatively, significantly associated with actively seeking medical care (Table 7). Employing traditional medicine was negatively, significantly associated with not recognizing the biting dog (aOR = 0.03, 95% C.I. 0.00, 0.82) for this sub-population. While increased poverty level (aOR = 1.14, 95% C.I. 0.99, 1.31) and living greater than 20 kilometers from a location where PEP could be received (aOR = 0.16, 95% C.I. 0.03, 1.00) in the first model as well as recognizing the biting dog as a neighbor’s dog (aOR = 0.13, 95% C.I. 0.01, 1.25) in the second model showed p-values less than the cutoff of 0.1, the range in confidence intervals prevents the association from being considered significant. The outcome of receiving PEP was not able to be modelled since only 3 bite victims reported this behavior after a dog bite.

**Table 7:** Characteristics associated with healthcare-seeking behaviors among 100 canine-bite victims, Uganda, 2013.

		Outcome: Sought medical care (n = 56)		Outcome: Employed traditional medicine (n = 21)	
Variables in model	n^a^	aOR (95% C.I.)	p-value	aOR (95% C.I.)	p-value
**Age**	19	0.98 (0.95–1.01)	0.16	1.00 (0.96–1.04)	0.86
**Sex**					
Male	52	ref		ref	
Female	48	0.40 (0.13–1.18)	0.10	2.58 (0.64–10.44)	0.18
**% below poverty**	47.1	1.14 (0.99–1.31)	0.07	0.93 (0.79–1.09)	0.34
**District**					
Bundibugyo	11	ref		ref	
Kabarole	43	0.53 (0.06–4.29)	0.55	0.22 (0.02–2.38)	0.21
Kampala	8	73.90 (0.13–41792.40)	0.18	0.04 (0.00–63.56)	0.40
Mbale	20	2.03 (0.22–18.90)	0.53	0.00 (0.00 - >1.0E12)	0.96
Wakiso	18	19.49 (0.42–912.80)	0.13	0.10 (0.00–7.02)	0.29
**Ownership of biting dog**					
Own dog	9	ref		ref	
Neighbor’s dog	44	1.70 (0.29–9.86)	0.55	0.13 (0.01–1.25)	0.08
Community dog	33	2.22 (0.35–14.13)	0.40	0.16 (0.02–1.75)	0.13
Did not recognize dog	14	1.19 (0.14–10.02)	0.87	0.03 (0.00–0.82)	**0.04**
**Distance to PEP**					
0–5 km	53	ref		ref	
6–20 km	22	0.74 (0.23–2.44)	0.62	1.81 (0.34–9.49)	0.48
>20 km	8	0.16 (0.03–1.00)	0.05	5.00 (0.72–34.72)	0.10
Unknown/Unreported	17	0.18 (0.03–0.99)	**0.05**	2.78 (0.30–25.80)	0.37
**Rabies exposure risk**					
Low	17	ref		ref	
Medium	65	0.91 (0.22–3.68)	0.89	3.83 (0.46–31.85)	0.21
High	18	1.25 (0.21–7.59)	0.81	2.78 (0.22–35.44)	0.43
**Wealth score**	0.9	1.02 (0.97–1.06)	0.52	0.97 (0.91–1.03)	0.32

aOR, adjusted odds ratio; C.I., confidence interval

^a^Averages for continuous variables and counts otherwise

### Discordance among attitudes and practices within the study population

Overall, about half as many respondents who had not been bitten said they would wash their wound if bitten compared to bite victims who reported washing their bite wound (Table 4). Twenty-one percent of bite victims consulted a traditional healer, though only 1.6% of not bitten respondents indicated that they would consult with a traditional healer if bitten. No bite victims contacted a medical doctor though 6.9% of not bitten respondents indicated that they would. Thirteen percent of bite victims reported that they did nothing once bitten, though only 2.1% of not bitten respondents indicated they would do nothing if bitten. A comparable amount of respondents from both sub-populations did or said they would call a veterinarian, seek medical treatment, receive rabies PEP, or isolate the dog or submit it for testing.

## Discussion

Understanding barriers to appropriate wound care and to effectively seeking healthcare after a canine-bite event, or potential rabies exposure, is necessary to reduce the overall burden of dog-mediated human rabies deaths within a population. Our findings reveal a varying inconsistency among what non-bite victim study participants in Uganda said they would do if potentially exposed to rabies and what reported canine-bite victims actually did in response to exposures. Additionally, our findings suggest that increased knowledge about rabies disease, transmission, and post-exposure care could potentially improve post-exposure outcomes.

This study was part of a 3-country Congo-basin survey effort, also including Cameroon and the Democratic Republic of the Congo, to improve understanding of barriers to rabies elimination. Like results from the not bitten respondents reported here, a similar KAP study conducted in Cameroon found that increased rabies knowledge was associated with increased odds that an individual would seek medical care and receive PEP after a potential bite exposure [11]. Other KAP studies conducted throughout East Africa have reported similar findings on knowledge of rabies disease and post-exposure treatment as reported here [17, 18]. Authors of a rabies KAP study conducted in Tanzania concluded that those who had greater education were more likely to have greater knowledge about rabies [16]. Additionally, in Northwest Ethiopia, a strong association was found between KAP scores and education level, but authors concluded that a stronger form of rabies-specific education was necessary to improve knowledge of rabies transmission, symptoms, and more [18]. Etheart et al. reported that implementation of community-based surveillance and counseling provided by integrated bite case management programs in Haiti were the most effective methods in changing healthcare-seeking behaviors of bite victims [19]. It is possible that implementation of similar programs in Uganda would improve community knowledge, increase favorable healthcare-seeking behaviors, and reduce subsequent human rabies deaths. Therefore, investments in rabies education could increase the frequency of bite victims appropriately washing the wound and seeking post-bite medical care and PEP.

While seeking traditional medicine may not be inherently harmful, not seeking medical treatment in combination with traditional treatments is risky. In this survey, seeking traditional medicine was the second highest reported healthcare-seeking behavior among bite victims. Model results presented in Table 7 indicated that seeking traditional medicine after a bite event was negatively associated with bite victims who were bitten by dogs they did not recognize. This association shows promise in the decision-making abilities of the bite victims (14%) that fall into this category, particularly since those bitten by an unrecognized dog could not inquire about its vaccination history. However, a previously published paper on the same cohort discovered that a larger number of dogs were owned by individuals who resided in impoverished communities and that dog vaccination coverage was much lower in these communities compared to wealthy, high population density communities [10]. These concerning findings suggest conditions are present in the surveyed areas that could lead to increased risk of dog-mediated human rabies transmission in high-poverty communities. The fact that 4 of the 5 suspected human rabies deaths identified in this study were from impoverished communities further supports this hypothesis. Additionally, though the cause of death is unknown for all canine-bite victims included in this analysis, the finding that all post-bite deaths were among unvaccinated persons lends support to the important role PEP plays in preventing dog-mediated human rabies deaths, and the low utilization of PEP among bite victims in this survey warrants further investigation into community awareness and access to appropriate post-bite treatment.

Rates of actively seeking medical care and receiving PEP after being bitten were higher among those who reported more bite events with a higher suspicion of a rabies exposure, indicating that individual self-risk-assessment may have played a role in choosing healthcare-seeking behaviors. This behavior was also found in a study conducted in Haiti in 2015, in which the authors found that the odds of receiving a vaccine were more than 8 times greater for bite victims with higher rabies exposure risk scores [20]. Additionally, the uncertain distribution and availability of PEP, particularly at the time of the survey in 2013, may serve as an important barrier to initiating PEP after a potential rabies exposure regardless of wealth status. This is of particular interest since only 3 of the bite victims received PEP though 56 sought care after exposure. The reason for bite victims not receiving PEP after seeking medical care was not recorded in the survey. Though the state of rabies surveillance, prevention, and control is not well established for the year during which this survey was conducted, Uganda’s Annual Health Sector Performance Report cites that progress towards rabies prevention and control in the years 2012 and 2013 included the dissemination of informational materials such as pamphlets and flyers, training of data managers for surveillance of various zoonotic diseases including rabies, and training of health staff on zoonotic disease investigation, prevention, and control [21]. It is possible that community-level variables not considered in this analysis may lend to patterns in healthcare-seeking behaviors, though these variables are not evident in Uganda-specific documentation from the time of this survey [21]. Unsurprisingly, bite victims who reported not knowing of a location where rabies PEP could be received were less likely to seek medical care after an exposure. This finding reveals the need for increased accessibility of rabies PEP, increased rabies awareness in these communities, and for exposed individuals to be educated on where to receive treatment.

Comparing the attitudes and practices of the survey population regarding post-exposure healthcare-seeking behaviors aids in determining factors that are identifiable by the study population, as well as underlying factors not-identifiable by the study population, which influence healthcare-seeking behaviors. Compared with the responses from people who had not been bitten reporting what they would theoretically do if bitten by a dog, a smaller proportion of bite victims sought medical treatment and a higher proportion consulted with a traditional healer. This discrepancy between what individuals in the study population said they would do if bitten by a dog compared to what individuals in the study population actually did when bitten by a dog may have harmful implications on human health and policy, as models using theoretical health actions or responses may not be accurate. If rabies control programs are developed and implemented based on falsely suggested theoretical health actions, they may not be effective in reality, and may end up wasting time and resources while not improving adverse health outcomes or changing unfavorable healthcare-seeking behaviors. Additionally, it is crucial that traditional healers be educated to refer bite victims to receive PEP, and that all traditional and non-traditional healers and healthcare workers receive continuous training on post-exposure practices. Incorporating traditional healers and traditional healing methods into rabies control programs may increase the acceptance of these programs within communities and improve PEP seeking behaviors. This is increasingly relevant to realizing the goal of eliminating canine-mediated human rabies deaths by 2030, which requires that healthcare systems in endemic countries such as Uganda be efficient in providing rabies PEP [6].

Rates of dog bites vary greatly between rabies endemic countries as well as among high- and low-income localities within countries [4, 15, 22]. The overall annual community rate of canine-bites for the entire study population was 2.3 per 100 people (2.3%). Of all the households interviewed, 11.8% reported that someone at the residence had been bitten by a dog in the past year. In other studies with similarly designed KAP surveys and research goals, a range of 5.3% to 31.3% of randomly selected entities (respective households or individuals) reported canine-bite events in varying locations around the world[11, 23–26]. The particular phrasing or translation of the question in each individual study’s survey may play a role in the difference in rates of canine-bites reported by the households or individuals. In the cases of other surveys, some report the number of bite events within a particular timeline while others pose no timeline for reporting bite incidences, making numbers and rates of canine-bites difficult to directly compare between surveys. A more recent KAP survey conducted in the Mbale district of Uganda concluded that of the survey respondents, only 44% has sufficient knowledge of rabies, and authors suggest that, even currently, more educational awareness regarding rabies is needed in Mbale [27]. Findings from another recent KAP survey conducted in the Moyo and Ntoroko districts of Uganda suggest that dog bites are still high, with 75% and 62.5% of respective bite rates [28]. Also, in East Africa, a 2015 KAP study from Ethiopia reports a bite rate of 42% among survey respondents [22]. Though the dog bite rate presented in this KAP survey is relatively low, higher and more recently calculated dog bite rates, in combination with reports of low rabies knowledge, indicate that potential exposure to rabies in dog bites continues to exist, specifically in the largely endemic East Africa.

While KAP studies are useful tools for data collection, they are hypothesis generating surveys, and many of the associations revealed here warrant further investigation [29–31]. Since the plan for achieving the 2030-elimination goal focuses primarily on the effective use of and access to human vaccinations [6, 32], knowledge of post-exposure care remains essential in preventing canine-mediated human rabies deaths pending the effective establishment of surveillance systems and bite case management programs in endemic countries such as Uganda. Additionally, preventative measures of wound washing and dog vaccination are equally critical and should be stressed during educational or vaccination campaigns [33]. High rates of animal bites were continually reported between 2001 and 2015, and in 2018 Masiira et al. concluded that human exposures to rabid animals remain a serious public health threat [8]. Since the time of survey administration in 2013, Uganda published a National One Health Strategic Plan encouraging and planning for the prioritization of neglected zoonotic diseases, including rabies, though it is unclear if any capacity-building has been realized since [34]. In the same year, the non-government organizations ‘Global Alliance for Rabies Control,’ or GARC, and ‘The Big Fix’ both report recent efforts to offer free and accessible dog vaccinations in Uganda, though the widespread and total effects of these efforts is unknown (https://rabiesalliance.org/resource/collaborative-control-efforts-uganda, http://thebigfixuganda.org/rabies-in-uganda.html). Many vaccination campaigns that occur in sub-Saharan Africa have not achieved 70% vaccination coverage in dogs, as the sub-Saharan environment requires a specific methodology different from other parts of the world which have shown successes in such campaigns [35, 36] In the future, even if the 70% canine vaccination goal is reached throughout Uganda, dog bite events will never cease to occur, and it will remain essential that individuals are aware of the dangers of rabies and how to properly seek post-exposure care [6].

Possible limitations of this analysis include recall or information bias for self-reported exposures, which could result in differential or non-differential misclassification, biasing the results in either direction. Particularly, the ‘distance to nearest PEP’ variable, or the nearest distance from the survey respondent’s residence where rabies PEP could be received, represents a perceived distance and not an actual distance. Since all symptoms of reported illnesses resulting from dog bites were not recorded, we are unable to determine if the reported deaths due to these illnesses were indeed from CRVV infection. Respondents may not have answered questions truthfully if they felt pressured to give “correct” answers. However, the language used in the questionnaire was designed to prevent this. Additionally, the gap in time between survey administration and data analysis may reduce the applicability of findings.

There are other possible limitations to the study that may have prevented our models from identifying barriers to particular healthcare-seeking behaviors after a bite event for the bite victims. It is possible that the factors which drive individuals to seek medical attention and PEP are not different among the sub-populations of not bitten respondents and canine-bite victims. Other factors may play a role in this type of independent, risk-assessment decision making which would be difficult to capture in a survey of this kind. It is also possible that, besides independent motivation to seek medical attention, receiving PEP is a factor of the individual’s medical provider or facility instead of a personal decision.

## Conclusion

The results from this study highlight the impact of the absence of a structured rabies disease surveillance system or rabies prevention and control program in a sub-Saharan country, as was the state of Uganda’s rabies surveillance, prevention, and control capacities at the time of this KAP survey. There was varying discordance among what not bitten respondents said they would do if bitten by a dog and behaviors exhibited by canine-bite victims. As expected, increased rabies knowledge was associated with a non-bitten respondent indicating that they would seek medical care and receive PEP after a potential rabies exposure, highlighting the importance of awareness of the risks for rabies in Uganda. Previously established educational platforms may be helpful, but consideration of more widespread and effective awareness programs, complemented by robust community-led rabies surveillance programs, that effectively incorporate traditional methods may help show communities that the disease is a real and present danger, worthy of adherence to preventive messaging.

## Supporting information

S1 Appendix Survey questionnaire. (DOCX)

S1 Table Characteristics of villages among the 798 surveyed households, Uganda, 2013. (DOCX)

S2 Table Assessment of domicile features. (DOCX)
