# Supplementary material for: Rabies post-exposure healthcare-seeking behaviors and perceptions: Results from a knowledge, attitudes, and practices survey, Uganda, 2013
Source: PLoS One. 2021 Jun 2;16(6):e0251702. doi: 10.1371/journal.pone.0251702 (PMC8171952; doi:10.1371/journal.pone.0251702)
Supplement: S1 Appendix — (DOCX) [file pone.0251702.s001.docx]

S1 Appendix. Survey questionnaire.

1. Interview Date:

2. Interviewer:
*Participant Demographic Information*

3. Consent obtained (Note: Form requests confirmation of adult age)

a. Yes

b. No

*4. How old are you?

*5. Gender

a. Male

b. Female

c. Other
d. Decline to answer

*6. How many years of schooling have you completed?

*7. How many people live in your household?

8. How many children below the age of 18 live in your household?

*Participant Housing Description*

9. How many years have you lived in this place?

*10. (Surveyor assistant - observe and describe construction of house).

a. Floor – cement / tile / soil / wood / other:

b. Walls – cement / metal / mud / straw or palm leaves / other:

c. Roof – cement / metal / straw or palm leaves / other:

d. Windows – none / metal / curtain / glass / other:

e. Door – none / metal / curtain / wood / other:

*Owned Animals*

*11.What kind of livestock does your family own? How many head of each? Mark all that apply.

a. None

b. Chickens, indicate number

c. Cattle, indicate number

d. Goats, indicate number

e. Sheep, indicate number

f. Other: (free response, indicate frequency)

g. Declined to answer

*12. Does your family currently own any dogs? If yes, how many? (if answer is No, skip to 18)

a. No

b. Yes, indicate number

c. Declined to answer

13. What are the ages of your dogs in years?

a. Free response

14. What best describes the amount of time that your dog(s) spends indoors?

a. Never

b. Infrequently

c. Occasionally

d. Frequently

e. Always

f. Declined to answer

15. What level of care do you provide for your dog(s)? Mark all that apply.

a. None

b. Food

c. Water

d. Shelter

e. Veterinary Care

f. Other: (free response, indicate frequency)

g. Declined to answer

16. Have any of your dog(s) been vaccinated against rabies?

a. Yes, indicate number

b. No

c. I don’t know

d. Declined to answer

17. If any of your dog(s) have not been vaccinated for rabies, what is the reason?

a. Dog is too young

b. No money to buy vaccine

c. No vaccine available

d. No need to vaccinate

e. Other: (free response, indicate frequency)

f. Declined to answer

18. In the past five years, have you owned any dogs that died?

a. No

b. Yes, indicate number

c. Declined to answer

19. For the dogs that died, what was the cause of death? Indicate frequency of each if more than one dog.

a. Accident/injury

b. Disease/illness

c. Other: (free response, indicate frequency)

d. I don’t know

e. Declined to answer

20. Does your family care for any dogs in the community? If yes, how many? (if answer is No, skip to 22)

a. No

b. Yes, indicate number

c. Declined to answer

21. What level of care do you provide for the community dog(s)? Mark all that apply.

a. None

b. Food

c. Water

d. Shelter

e. Veterinary Care

f. Other: (free response, indicate frequency)

g. Declined to answer

*Dog Bite Information*

*22. Have you or anyone in the household been bitten by a dog? Mark all that apply. (if answer is No, skip to 34)

a. No

b. Yes, me

c. Yes, an adult family member (indicate number if more than one)

d. Yes, my child (indicate number if more than one)

e. Declined to answer

*23. For each person identified, what is their gender?

a. Male

b. Female

c. Other

d. Decline to answer

*24. For each person identified, how old were you/they when bitten by the dog?

*25. For each person identified, on how many separate occasions were you/they bitten by a dog? Mark all that apply and indicate frequency if multiple persons were identified.

a. One occasion

b. Two occasions

c. Three occasions

d. Four occasions

e. Five occasions

f. More than five occasions

*26. For each person identified, where were you/they when you were bitten by the dog? Mark all that apply and indicate frequency if multiple persons were identified.

a. At home

b. Not at home, but within local community

c. Outside of local community

d. Declined to answer

*27. For each person identified, what were you doing when you/they were bitten the dog? Mark all that apply and indicate frequency if multiple persons were identified.

a. At home, unprovoked attack by own dog

b. At home, unprovoked attack by community dog

c. Playing with, restraining or feeding the dog

d. Playing with, restraining of feeding puppies of the (bitch) dog

e. Visiting the dog’s home

f. Walking in community, avoiding the dog

g. Herding livestock, avoiding the dog

h. Hunting wild animals, avoiding the dog

i. Playing or recreating outdoors, avoiding the dog

j. Other: (free response)

k. Declined to answer

*28. For each person identified, where on your body were you/they bitten by the dog? Mark all that apply and indicate frequency if multiple persons were identified.

a. Head/face

b. Torso/trunk

c. Hands/feet

d. Arm

e. Leg

f. Other: (free response, indicate frequency)

g. Declined to answer

*29. For each person identified, what did you/they do when bitten by the dog? Mark all that apply and indicate frequency if multiple persons were identified.

a. Nothing

b. Washed wound

c. Consulted with a traditional healer

d. Call a medical doctor

e. Call a veterinarian

f. Actively sought medical treatment at a pharmacy, hospital, clinic or outpost

g. Received rabies post-exposure prophylaxis

h. Isolated the dog for observation

i. Submitted dog for disease testing

j. Killed the dog

k. Killed and ate the dog

l. Other: (free response, indicate frequency)

m. Declined to answer

30. (If answer to 29 was ‘f’ or ‘g’) What was the amount of time between when you/they were bitten and medical treatment was sought? Mark all that apply and indicate frequency if multiple persons were identified.

a. < 1 day

b. 1-3 days

c. 4-6 days

d. 1-2 weeks

e. 3-4 weeks

f. 5-8 weeks

g. > 2 months

h. Other: (free response, indicate frequency)

i. Declined to answer

*31. For each person identified, did you/they develop illness due to the bite?

a. Yes

b. No

c. Unknown

d. Decline to answer

*32. For each person identified, did you/they die due to illness caused by the bite?

a. Yes

b. No

c. Unknown

d. Decline to answer

*33. For each person identified, how familiar were you/they with the dog? Mark all that apply and indicate frequency if multiple persons were identified.

a. Own (family) dog

b. Neighbor’s dog

c. Dog in community

d. Did not recognize dog

e. Declined to answer

*34. (if never been bitten by a dog) What would you do if you were bitten by a dog that you recognize or own? Mark all that apply.

a. Nothing

b. Wash wound

c. Consult with a traditional healer

d. Call a medical doctor

e. Call a veterinarian

f. Actively seek medical treatment at a pharmacy, hospital, clinic or outpost

g. Receive rabies post-exposure prophylaxis

h. Isolate the dog for observation

i. Submit dog for disease testing

j. Kill the dog

k. Kill and eat the dog

l. Other: (free response)

m. Declined to answer

*35. (if never been bitten by a dog) What would you do if you were bitten by a dog that you do not recognize or own? Mark all that apply.

a. Nothing

b. Wash wound

c. Consult with a traditional healer

d. Call a medical doctor

e. Call a veterinarian

f. Actively seek medical treatment at a pharmacy, hospital, clinic or outpost

g. Receive rabies post-exposure prophylaxis

h. Isolate dog for observation

i. Submit animal for disease testing

j. Kill the dog

k. Kill and eat the dog

l. Other: (free response)

m. Declined to answer

36. If you saw a dog in your village that looked sick, what would you do? Mark all that apply.

a. Nothing

b. Call local authorities

c. Call a friend

d. Avoid the animal

e. Scare (shoo) animal away

f. Kill the dog

g. Kill and eat the dog

h. Submit the animal for disease testing

i. Other: (free response)

j. Declined to answer

*Domestic Animal Illness Questions*

37. Does your family currently own any cats? If yes, how many?

a. No

b. Yes, indicate number

c. Declined to answer

38. Have you or anyone in this household had illness that was attributed to a pet/livestock animal bite? Mark all that apply. (if answer is No, skip to 42)

a. No

b. Yes, me

c. Yes, an adult family member (indicate number if more than one)

d. Yes, my child (indicate number if more than one)

e. Declined to answer

39. If the answer to 38 was yes, what kind of animals were thought to have caused the illness? Mark all that apply and indicate frequency if multiple persons were identified.

a. Dog

b. Cat

c. Cattle

d. Goat

e. Sheep

f. Pig

g. Chickens

h. Other: (free response, indicate frequency)

i. Unknown

j. Decline to answer

40. If the answer to 38 was yes, has anyone in your household died as a result of an animal bite?

a. Yes

b. No

c. Unknown

d. Decline to answer

41. If the answer to 38 was yes, what were the symptoms? Mark all that apply and indicate frequency if multiple persons were identified.

a. Skin rash/discoloration/ infection

b. Unusual bleeding (e.g. from nose/mouth)

c. Hypersalivation

d. Fever

e. Cough

f. Sneezing

g. Runny nose

h. Chest congestion

i. Muscle pain

j. Difficulty breathing

k. Headache

l. Convulsions

m. Altered mental state (dementia)

n. Unconsciousness/coma

o. Muscle weakness/paralysis

p. Vomiting or diarrhea or stomach cramps

q. Miscarriage/stillbirth

r. Death

s. Multiple persons

t. Other: (Free response)

u. Declined to answer

*Wild Animal Bite Information*

42. Have you or anyone in this household been bitten by a wild animal (including rats)? Mark all that apply. (if answer is No, skip to 53)

a. No

b. Yes, me

c. Yes, an adult family member (indicate number if more than one)

d. Yes, my child (indicate number if more than one)

e. Declined to answer

43. For each person identified, how old were you/they when you were bitten by the wild animal?

44. For each person identified, did you/they develop illness attributed to that animal bite? (if answer is No, skip to 46)

a. No

b. Yes, me

c. Yes, an adult family member (indicate number if more than one)

d. Yes, my child (indicate number if more than one)

e. Declined to answer

45. For each person identified, what were the symptoms of the illness attributed to the animal bite? For each person identified, what were the symptoms? Mark all that apply and indicate frequency if multiple persons were identified.

a. Skin rash/discoloration/ infection

b. Unusual bleeding (e.g. from nose/mouth)

c. Hypersalivation

d. Fever

e. Cough

f. Sneezing

g. Runny nose

h. Chest congestion

i. Muscle pain

j. Difficulty breathing

k. Headache

l. Convulsions

m. Altered mental state (dementia)

n. Unconsciousness/coma

o. Muscle weakness/paralysis

p. Vomiting or diarrhea or stomach cramps

q. Miscarriage/stillbirth

r. Death

s. Multiple persons

t. Other: (Free response)

u. Declined to answer

46. For each person identified, what kind of wild animal was it? Mark all that apply and indicate frequency if multiple persons were identified.

a. Jackal

b. Hyena

c. Mongoose

d. Honey badger

e. Monkey or other primate

f. Fox

g. Bat

h. Rat

i. Other: (free response)

j. I don’t know

k. Declined to answer

47. For each person identified, on how many separate occasions were you/they bitten by a wild animal? Mark all that apply and indicate frequency if multiple persons were identified.

a. One occasion

b. Two occasions

c. Three occasions

d. Four occasions

e. Five occasions

f. More than five occasions

48. For each person identified, where were you/they when bitten by the wild animal? Mark all that apply and indicate frequency if multiple persons were identified.

a. At home

b. Not at home, but within local community

c. Outside of local community

d. Declined to answer

49. For each person identified, what were you doing when you were bitten by the wild animal? Mark all that apply and indicate frequency if multiple persons were identified.

a. In home, the animal entered home

b. Walking in community, avoiding the animal

c. Playing with, restraining or feeding the animal

d. Herding livestock, avoiding the animal

e. Hunting other animals

f. Hunting the animal

g. Playing or recreating outdoors, avoiding the animal

h. Other: (free response)

i. Declined to answer

50. For each person identified, where on your body were you/they bitten by the wild animal? Mark all that apply and indicate frequency if multiple persons were identified.

a. Head/face

b. Torso/trunk

c. Hands/feet

d. Arm

e. Leg

f. Other: (free response)

g. Declined to answer

51. For each person identified, what did you do after you/they were bitten by the wild animal? Mark all that apply and indicate frequency if multiple persons were identified.

a. Nothing

b. Washed wound

c. Consulted with a traditional healer

d. Call a medical doctor

e. Call a veterinarian

f. Actively sought medical treatment at a pharmacy, hospital, clinic or outpost

g. Received rabies post-exposure prophylaxis

h. Isolated the animal for observation

i. Submitted animal for disease testing

j. Killed the animal

k. Killed and ate the animal

l. Other: (free response)

m. Declined to answer

52. (If answer to 51 was ‘f’ or ‘g’) For each person identified, what was the amount of time between when you/they were bitten and when medical treatment was sought? Mark all that apply and indicate frequency if multiple persons were identified.

a. < 1 day

b. 1-3 days

c. 4-6 days

d. 1-2 weeks

e. 3-4 weeks

f. 5-8 weeks

g. > 2 months

h. Other: (free response)

i. Declined to answer

53. (If never been bitten by a wild animal) If you were bitten by a wild animal, what would you do? Mark all that apply.

a. Nothing

b. Wash wound

c. Consult with a traditional healer

d. Call a medical doctor

e. Call a veterinarian

f. Actively seek medical treatment at a pharmacy, hospital, clinic or outpost

g. Receive rabies post-exposure prophylaxis

h. Isolate the animal for observation

i. Submit animal for disease testing

j. Kill the animal

k. Kill and eat the animal

l. Other: (free response)

m. Declined to answer

54. If you saw a wild animal in your village that looked sick, what would you do? Mark all that apply.

a. Nothing

b. Call local authorities

c. Call a friend

d. Avoid the animal

e. Scare (shoo) animal away

f. Kill the animal

g. Kill and eat the animal

h. Submit the animal for disease testing

i. Other: (Free response)

j. Declined to answer

*Rabies Knowledge Section*

55. How much do you know about a disease called rabies? Note: interviewer must evaluate.

a. I have never heard of rabies

b. Little knowledge (i.e., have heard of rabies/dog disease, but can’t identify transmission routes or severity of disease)

c. Basic understanding (knowledge that rabies is both a highly fatal disease and is transmitted by dog bite)

d. Extensive knowledge (basic understanding plus knowledge of non-bite routes of exposure AND wildlife reservoirs besides dogs without prompting)

e. Declined to answer

*56. How severe is the disease called rabies?

a. Mild

b. Somewhat severe

c. Very severe, but possible to recover

d. Very severe, resulting in death

e. I don’t know

f. Declined to answer

*57. How do humans get rabies from an infected animal? Mark all that apply.

a. Bite

b. Scratch

c. Observing the animal

d. Touching the animal

e. Contact with blood

f. Contact with saliva

g. Contact with urine/feces

h. Other: (free response)

i. I don’t know

j. Declined to answer

*58. What animals can be infected with rabies? Mark all that apply

a. Dogs

b. Cats

c. Livestock (Cattle, sheep, goats, etc.)

d. Poultry (Chickens, ducks, geese, etc.)

e. Horses

f. Jackals

g. Hyenas

h. Mongoose

i. Monkeys or other primate

j. Fox

k. Wild Birds

l. Bats

m. Rodents

n. Other: (free response)

o. I don’t know

p. Declined to answer

*59. If you thought that you had an exposure to an animal with rabies, what would you do?

a. Nothing

b. Wash wound

c. Consult with a traditional healer

d. Call a medical doctor

e. Call a veterinarian

f. Actively seek medical treatment at a pharmacy, hospital, clinic or outpost

g. Receive rabies post-exposure prophylaxis

h. Isolate the animal for observation

i. Submit animal for disease testing

j. Kill the animal

k. Kill and eat the animal

l. Other: (free response)

m. Declined to answer

60. Where do you normally go to receive medical treatment? Mark all that apply.

a. Veterinary Clinic

b. Pharmacy (Health Shop)

c. Medical Clinic

d. Hospital

e. Traditional Healer

f. Other: (free response)

g. Declined to answer

61. How far do you need to travel to receive medical care at this location? Indicate frequency if multiple locations were identified.

a. <1km

b. 1-5km

c. 6-10km

d. 11-20km

e. 21-30km

f. >30km

g. I don’t know

h. Declined to answer

*62. How far away is the location where you could receive rabies vaccination?

a. <1km

b. 1-5km

c. 6-10km

d. 11-20km

e. 21-30km

f. >30km

g. I don’t know

h. Declined to answer

63. Have you or anyone in this household ever received rabies vaccine? Mark all that apply and indicate frequency if needed (if answer is no, skip to 66)?

a. Yes, pre-exposure prophylaxis (inidcate frequency)

b. Yes, post-exposure prophylaxis (indicate frequency)

c. No

d. Declined to answer

64. (If answer to 63 is ‘a’ or ‘b’) Why did you or someone in your household receive rabies pre-exposure or post-exposure prophylaxis?

a. Pre exposure – free response: (identify any reason(s) that apply)

b. Post exposure – free response: (identify any reason(s) that apply)

c. Declined to answer

65. (If answer to 63 is ‘b’) What elements of post-exposure prophylaxis did you or someone in your household receive? Mark all that apply and indicate frequency if needed.

a. Rabies vaccine – Indicate number of doses (days) that treatment was administered

b. Rabies immune globulin (serum) Indicate number of doses (days) that treatment was administered (Note: should only be on Day 0)

c. Anti-tetanus serum - Indicate number of doses (days) that treatment was administered (Note: not part of rabies PEP, but may be commonly administered for bite wounds)

d. Other – free response

66. Where would (or did) you go to receive rabies post-exposure prophylaxis?

a. Pharmacy

b. Medical clinic

c. Traditional healer

d. Veterinary clinic

e. Hospital

f. Other: (free response)

g. Declined to answer

67. What are the primary obstacles for getting medical treatment in your community? Mark all that apply.

a. Lack of facilities to provide treatment

b. Lack of trained personnel at facilities to provide treatment

c. Lack of medicines at facilities for treatment

d. No means of transportation

e. No money to pay for treatment

f. Can’t miss work

g. Other: (free response)

h. I don’t know

i. Declined to answer

68. What do you know about veterinarians? Mark the best answer.

a. Person that provides care to sick or injured animals

b. Person that provides care to sick or injured humans

c. Person that provides care to sick or injured humans and animals

d. Person that provides education about animal health

e. Person that provides education about public health

f. Person that provides education about animal and public health

g. I don’t know or have never heard of a veterinarian

*Survey questions used for this analysis
