# Supplementary material for: Rabies post-exposure healthcare-seeking behaviors and perceptions: Results from a knowledge, attitudes, and practices survey, Uganda, 2013
Source: PLoS One. 2021 Jun 2;16(6):e0251702. doi: 10.1371/journal.pone.0251702 (PMC8171952; doi:10.1371/journal.pone.0251702)
Supplement: S1 Table — (DOCX) [file pone.0251702.s002.docx]

S1 Table. Characteristics of villages among the 798 surveyed households, Uganda, 2013.

| District | Village ID | Population Density  (per km^2^) | Distance to Urban Centre (km) | Percent Below Poverty (%) | Households Interviewed (Total Study Population Represented) | Number of Reported Dog Bites^a^ (Annual Community Bite Rate^b^) | Average Household Wealth Score^c^ (CI 95%)^d^ | Average Individual Knowledge Score^e^ (CI 95%)^d^ |
| --- | --- | --- | --- | --- | --- | --- | --- | --- |
| Kampala | | | | | | | 3.4 (1.8 – 5.1) | 12.2 (10.6 – 13.8) |
|  | AZ1 | 1401 | 0 | 5.7 | 38 (177) | 2 (1.1) | -2.1 (-4.5 – 0.3) | 10.8 (7.7 – 13.8) |
|  | CZ1 | 433 | 0 | 19.9 | 19 (109) | 0 (0.0) | 2.1 (1.0 – 9.2) | 10.1 (5.3 – 14.8) |
|  | KE1 | 2429 | 0 | 14.0 | 22 (94) | 2 (2.1) | 11.1 (7.2 – 15.2) | 15.7 (12.1 – 19.3) |
|  | KZ1 | 286 | 0 | 20.8 | 19 (108) | 2 (1.9) | 4.0 (1.1 – 7.0) | 10.5 (6.2 – 14.7) |
|  | MU1 | 34 | 2 | 20.6 | 12 (57) | 2 (3.5) | 3.3 (-3.4 – 10.0) | 16.3 (12.4 – 20.2) |
| Wakiso | | | | | | | 2.2 (0.8 – 3.7) | 7.9 (6.7 – 1.7) |
|  | BG2 | 3 | 14 | 39.7 | 17 (66) | 9 (13.6) | -1.0 (-5.4 – 3.3) | 5.5 (3.0 – 8.1) |
|  | MB2 | 41 | 14 | 39.2 | 29 (156) | 4 (2.6) | 4.0 (-0.4 – 8.4) | 7.4 (3.6 – 11.3) |
|  | BU2 | 31 | 15 | 40.0 | 34 (153) | 1 (0.7) | -0.2 (-5.3 – 4.9) | 6.5 (2.5 – 10.6) |
|  | KI2 | 41 | 10 | 37.9 | 29 (147) | 0 (0.0) | 4.3 (0.8 – 7.7) | 10.9 (7.7 – 14.1) |
|  | NC2 | 158 | 3 | 29.7 | 48 (178) | 4 (2.2) | 3.1 (0.8 – 5.3) | 8.5 (6.0 – 11.1) |
| Mbale | | | | | | | 0.7 (-0.3 – 1.7) | 8.8 (7.9 – 9.6) |
|  | BA3 | 34 | 2 | 59.5 | 60 (374) | 4 (1.1) | 0.9 (-1.2 – 3.0) | 11.9 (10.0 – 13.8) |
|  | BU3 | 57 | 18 | 56.0 | 52 (273) | 4 (1.5) | -1.1 (-3.3 – 1.1) | 7.0 (4.9 – 9.2) |
|  | KA3 | 633 | 0 | 52.9 | 33 (190) | 4 (2.1) | 4.5 (0.7 -8.3) | 9.5 (6.4 – 12.6) |
|  | MB3 | 49 | 4 | 61.5 | 33 (201) | 2 (1.0) | 1.1 (-2.5 – 4.7) | 6.3 (3.3 – 9.3) |
|  | NM3 | 441 | 0 | 52.9 | 71 (357) | 6 (1.7) | -0.2 (-2.6 – 2.1) | 8.2 (6.3 – 10.1) |
| Kabarole | | | | | | | 4.3 (2.8 – 5.8) | 15.8 (15.0 – 16.6) |
|  | BU4 | 10 | 1 | 52.7 | 31 (159) | 8 (5.0) | 0.3 (-2.6 – 3.3) | 15.5 (13.1 – 18.0) |
|  | KI4 | 226 | 1 | 51.4 | 30 (149) | 5 (3.4) | 6.5 (2.8 – 10.0) | 16.2 (14.2 – 18.1) |
|  | KK4 | 12 | 11 | 52.0 | 46 (259) | 16 (6.2) | 7.1 (3.6 – 10.6) | 15.1 (13.0 – 17.2) |
|  | NY4 | 447 | 1 | 49.2 | 37 (209) | 7 (3.3) | 3.3 (-1.0 – 7.6) | 16.6 (14.9 – 18.4) |
|  | RW4 | 43 | 25 | 56.4 | 23 (163) | 7 (4.3) | 2.7 (-0.3 – 5.8) | 15.8 (13.2 – 18.4) |
| Bundibugyo | | | | | | | -3.5 (-5.2 – -1.9) | 12.1 (10.7 – 13.5) |
|  | BB5 | 36 | 31 | 63.6 | 32 (231) | 1 (0.4) | -5.2 (-7.9 – -2.5) | 10.1 (7.2 – 13.0) |
|  | BG5 | 2 | 21 | 60.0 | 37 (234) | 4 (1.7) | -5.8 (-8.7 – -3.0) | 11.1 (8.5 – 13.8) |
|  | HK5 | 74 | 26 | 56.3 | 18 (117) | 5 (4.3) | -8.5 (-12.9 – -4.1) | 12.6 (8.5 – 16.7) |
|  | KY5 | 3 | 33 | 74.5 | 28 (214) | 1 (0.5) | 4.5 (1.3 – 7.7) | 15.4 (12.8 – 18.0) |
| Total | | 288.5 | 9.7 | 44.4 | 798 (4375) | 100 (2.3) | 1.5 (0.8 – 2.2) | 11.0 (10.4 – 11.6) |

^a^Number of reported dog bites within the 1 year from survey administration

^b^Rate per 100 people

^c^Average household wealth scored on education level of respondent, quality of construction of house, and livestock owned.

^d^One-way ANOVA yielded significant p-values for comparison of both average wealth and average knowledge scores between all districts.

^e^Average individual knowledge scored on responses to questions in the KAP survey designed to assess the surveyrespondent’s knowledge of the severity of rabies, transmission of disease, and attitudes concerning approaches to exposure.
