# Supplementary material for: Rabies post-exposure healthcare-seeking behaviors and perceptions: Results from a knowledge, attitudes, and practices survey, Uganda, 2013
Source: PLoS One. 2021 Jun 2;16(6):e0251702. doi: 10.1371/journal.pone.0251702 (PMC8171952; doi:10.1371/journal.pone.0251702)
Supplement: S2 Table — (DOCX) [file pone.0251702.s003.docx]

S2 Table. Assessment of domicile features.

|  | 1 (High) | 2 (Medium) | 3 (Low) | 4 (Poor) |
| --- | --- | --- | --- | --- |
| Floors | Cement and/or tile | Wood and/or brick | Soil | Cow dung, sand, and/or mud and trees |
| Walls | Cement and/or metal | Wood and/or brick | Stones, sand, and/or mud | Straw and/or palm leaves |
| Roof | Iron, metal, and/or roofing tiles | Cement | Wood | Straw and/or palm leaves (or banana fibers, grass) |
| Window | Glass and/ or metal | Wood | Curtains (or banana fibers, clothes, reeds, straw) | None |
| Door | Glass and/ or metal | Wood | Curtain and/or reeds | None |
